# Supplementary material for: Multiplex secretome engineering enhances recombinant protein production and purity
Source: Nat Commun. 2020 Apr 20;11:1908. doi: 10.1038/s41467-020-15866-w (PMC7170862; doi:10.1038/s41467-020-15866-w)
Supplement: Supplementary file 1 — Supplementary Information [file 41467_2020_15866_MOESM1_ESM.pdf]

Supplementary Material

Multiplex secretome engineering enhances recombinant protein production and purity

Kol et al. (2020)

Text to supplementary figure 1

A possible explanation for the HCP-reduced phenotypes observed in this study is that the creation of knockouts selects for faster-growing cells. As cells have go through a single-cell stage where survival rates are low, a selective pressure for better growth is imposed. It may thus be possible that the phenotype is not caused by specific knockouts, but is a more general effect of global downregulation of costly, secretory proteins. We quantified the HCP content of cell lines generated in an independent study addressing glycosylation[1]. These cell lines were subjected to 3 cycles of CRISPR-Cas9-mediated multiplex gene disruption. In the first round, 6 genes were disrupted. In the second round, two genes were disrupted. Finally, in the third round, another two genes were disrupted, resulting in a cell line harbouring 10 gene disruptions. No significant decrease in HCP content of WT CHO-S and the knockout cell lines is observed showing that the HCP-reduced phenotype is specifically caused by the removal of highly abundant proteins.

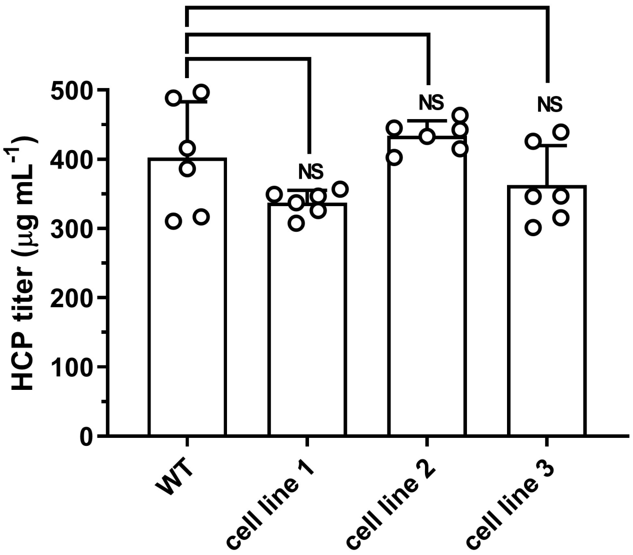

Supplementary Figure 1. HCP content of WT CHO-S and cell lines harbouring multiple gene disruptions. HCP content was measured of WT CHO-S (WT) and cell lines harbouring 6 gene disruptions (cell line 1), 8 gene disruptions (cell line 2), and 10 gene disruptions (cell line 3). HCP content was measured after cultivation for four days in shake flasks. Data are presented as mean values +/- SD (*n* = 6 independent experiments). Statistical analysis was performed using one-way ANOVA followed by Tukey's post-hoc test (ns = not significant). Source data are provided as a Source Data file.

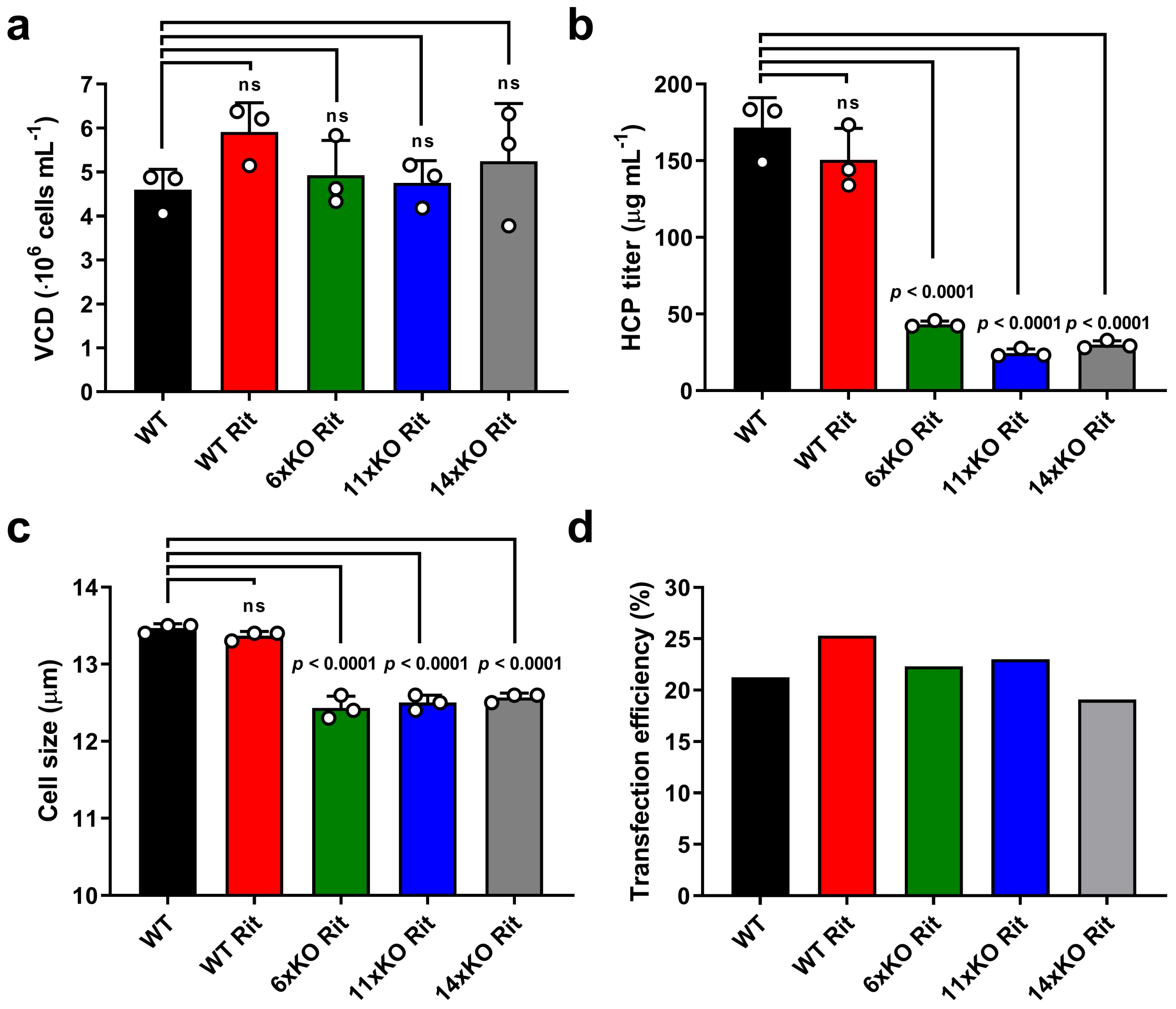

Supplementary Figure 2. VCD, HCP content, cell size and transfection efficiency. **a** VCD, **b** HCP content, **c** cell size, **d** transfection efficiency of WT (black), WT Rit (red), 6xKO Rit (green), 11xKO Rit (blue), and 14xKO Rit (grey) cell lines. All properties were measured after cultivation for four days in shake flasks. Data are presented as mean values +/- SD (n = 3 independent experiments) except for the transfection efficiency determination. Statistical analysis (**a**, **b**, **c**) was performed using one-way ANOVA followed by Tukey's post-hoc test (ns = not significant). Source data are provided as a Source Data file.

Text to Supplementary Figure 3

Growth characteristics and productivity of the knockout mutants were evaluated in an Ambr bioreactor using FortiCHO, ActiPro and OptiCHO media. Productivity was found to be highest in the 6xKO Rit cell line in OptiCHO medium (Supplementary Figure 3c, right panel). VCD of all clones cultured in this medium reached a maximum level of approximately 15 million cells per milliliter. While the VCD of WT Rit and 11xKO dropped after 8 days in culture, VCD of 6xKO Rit was constant for the remainder of the experiment (Supplementary Figure 3a, right panel). In addition, viability of the 6xKO Rit cell line remained higher than the other cell lines in this medium (Supplementary Figure 3b, right panel), indicating prolonged culture longevity. In ActiPro medium, we observed low VCD and productivity of both knockout cell lines as compared to WT Rit (Supplementary Figure 3a and 3c, center panels). In FortiCHO medium, the productivity of all cell lines was comparable, even though the cell density of WT Rit was higher than the 6xKO Rit and 11xKO Rit cell lines (Supplementary Figure 3a and 3c, left panels). Considerable differences in cell density, viability and titer were observed depending on the culture medium. These data show that high mAb production can be achieved in our knockout cell lines.

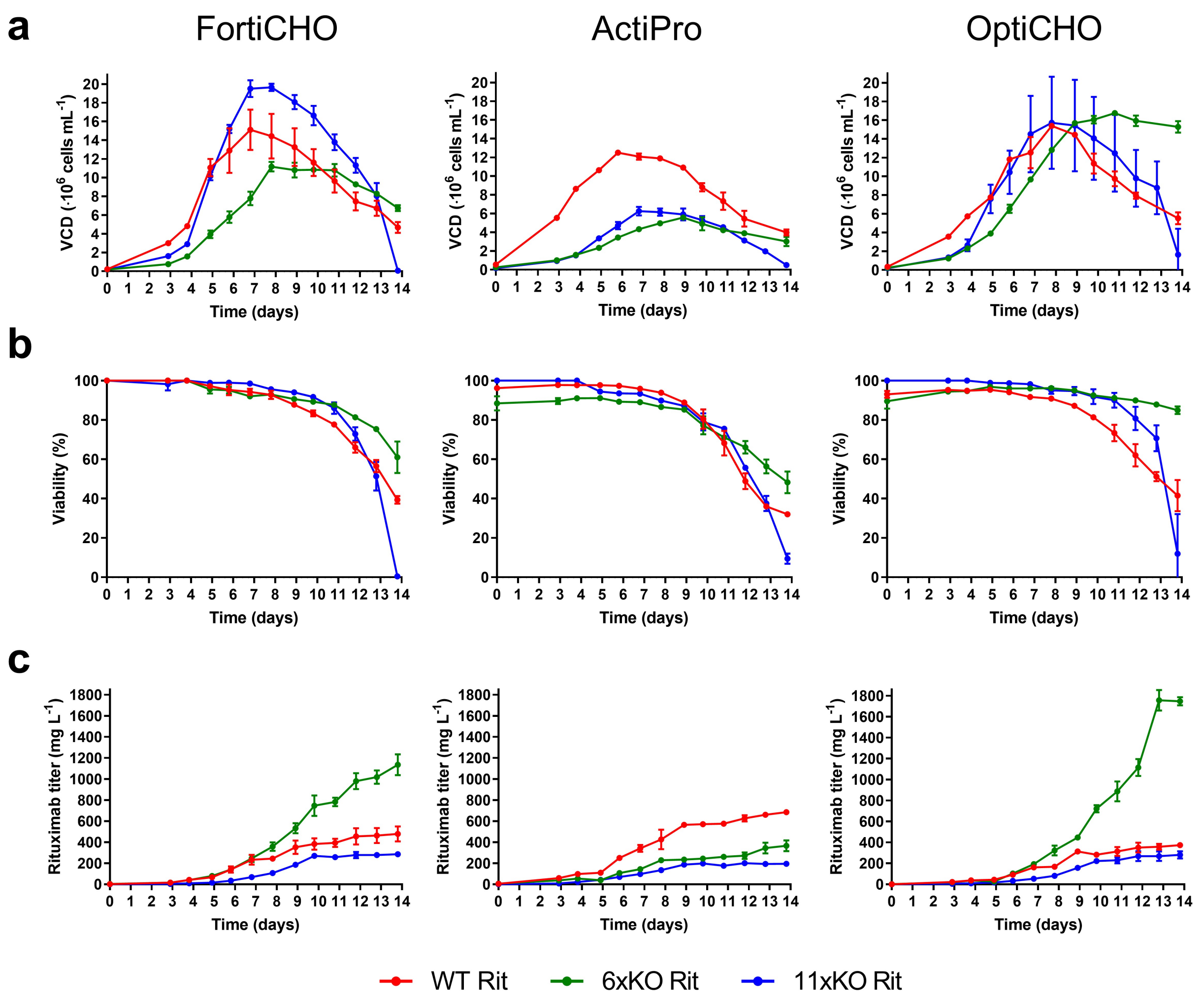

Supplementary Figure 3. Growth characteristics and productivity of the cell lines described in this study under fed-batch conditions in an Ambr bioreactor. **a** VCD, **b** viability, **c** Rituximab titer of WT Rit (red), 6xKO Rit (green), and 11xKO Rit (blue) cell lines measured daily during cultivation in an Ambr bioreactor. Cell lines were cultured in FortiCHO (left panels), ActiPro (center panels), and OptiCHO media (right panels). Data are presented as mean values +/- SD (*n* = 3 independent experiments). Source data are provided as a Source Data file.

Text to Supplementary Figure 4

Clonal variation can lead to a high degree of phenotypic heterogeneity among clones and can thus account for the observed HCP-reduced phenotype. Multiple antibody-producing knockout clones were therefore cultivated under fed-batch conditions, as well as one more wild-type clone. As these clones displayed lower mAb productivity than the original clones described in the main text, the calculated mAb/HCP ratio was generally lower than observed before. However, on average, all knockouts displayed improved purity over both wild-type clones tested, showing that the HCP-reduced phenotype is not caused by clonal variation

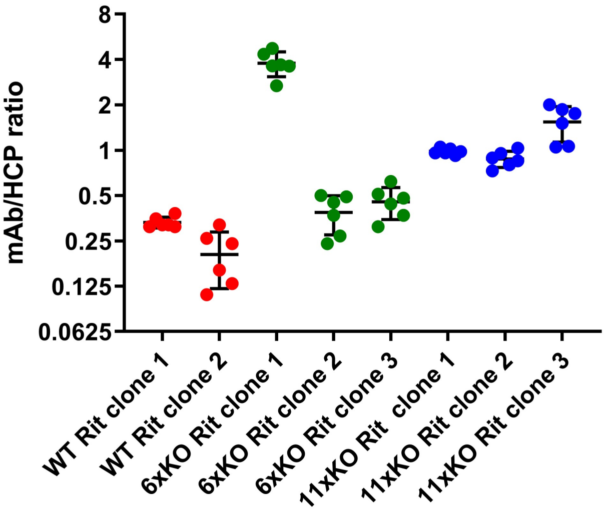

Supplementary Figure 4. Multiple antibody-producing knockout clones display the HCP-reduced phenotype under fed-batch cultivation in a DASGIP bioreactor. Clones of WT Rit (red), 6xKO Rit (green), and 11xKO Rit (blue) cell lines were grown in duplicate. WT Rit clone 1, 6xKO Rit clone 1 and 11xKO Rit clone 1 are technical replicates of the clones described in the main text, while all other clones are biological replicates. The HCP/mAb ratio was calculated by dividing the mAb titer by the HCP titer of the last three timepoints with viability above 90% in duplicate. Data is presented as mean values +/- SD (*n* = 6 independent samples). Source data are provided as a Source Data file.

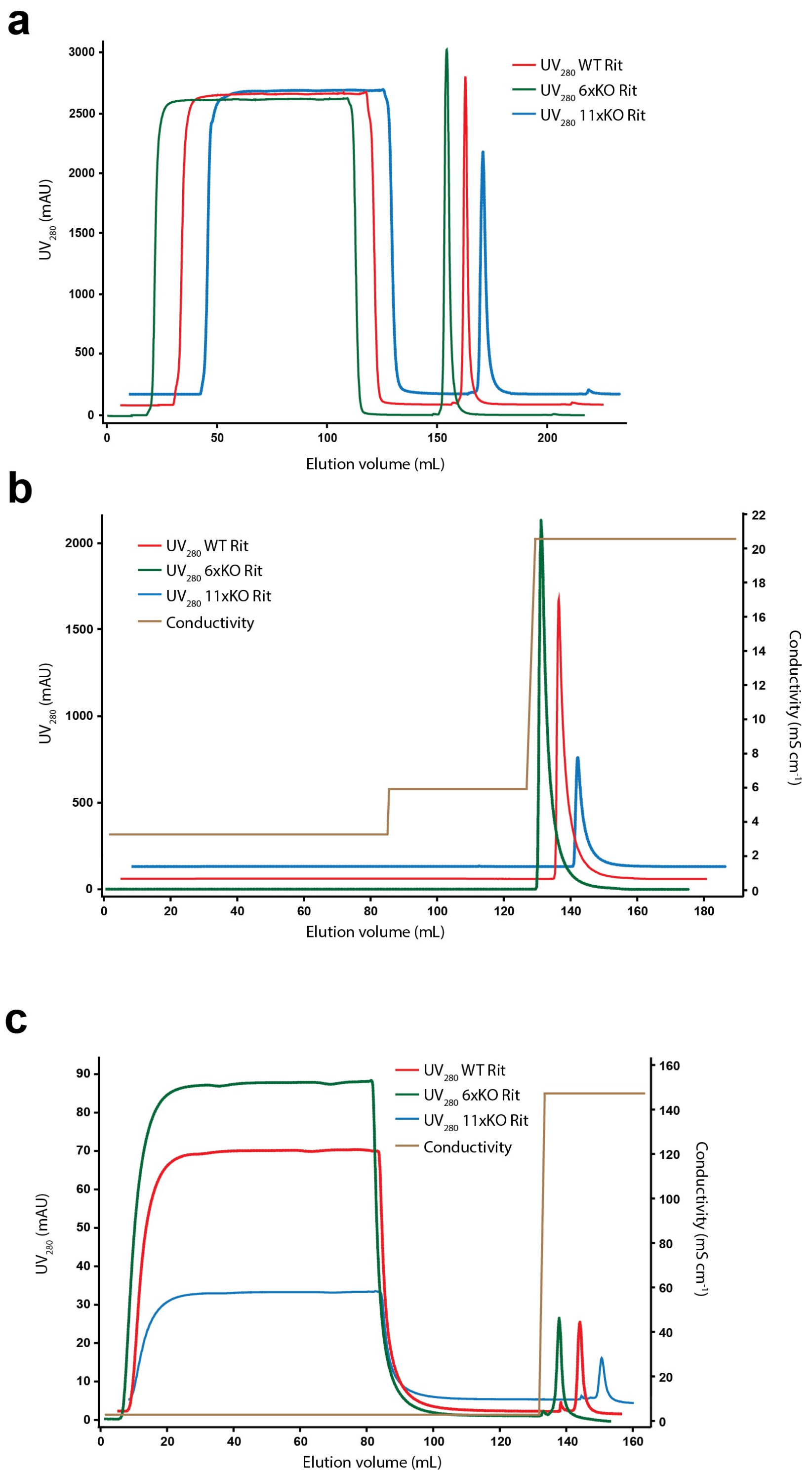

Supplementary Figure 5. Representative protein purification chromatograms. **a** Protein A, **b** CIEX and **c** AIEX chromatograms of the purification of Rituximab produced in WT Rit (red line), 6xKO Rit (green line) and 11xKO Rit (blue line) cell lines. Protein A and CIEX chromatography were run in bind-and-elute mode, while AIEX chromatography was run in flow-through mode. The conductivity profile during CIEX and AIEX is indicated (brown line).

Text to Supplementary Figure 6

Cell line engineering can have an effect on product quality attributes of recombinant proteins produced in these cells. There is a concern in our study that the HCP knockouts may have had an impact on the bioactivity and half-life of the model antibody Rituximab. We therefore analyzed the N-glycosylation pattern (Fig. S6a) and *in vitro* binding to cells expressing CD20 (Fig. S6b) of purified Rituximab. No changes were detected between wild-type CHO and the knockout cell lines in both assays, showing that the bioactivity and half-life of the mAb product are not perturbed.

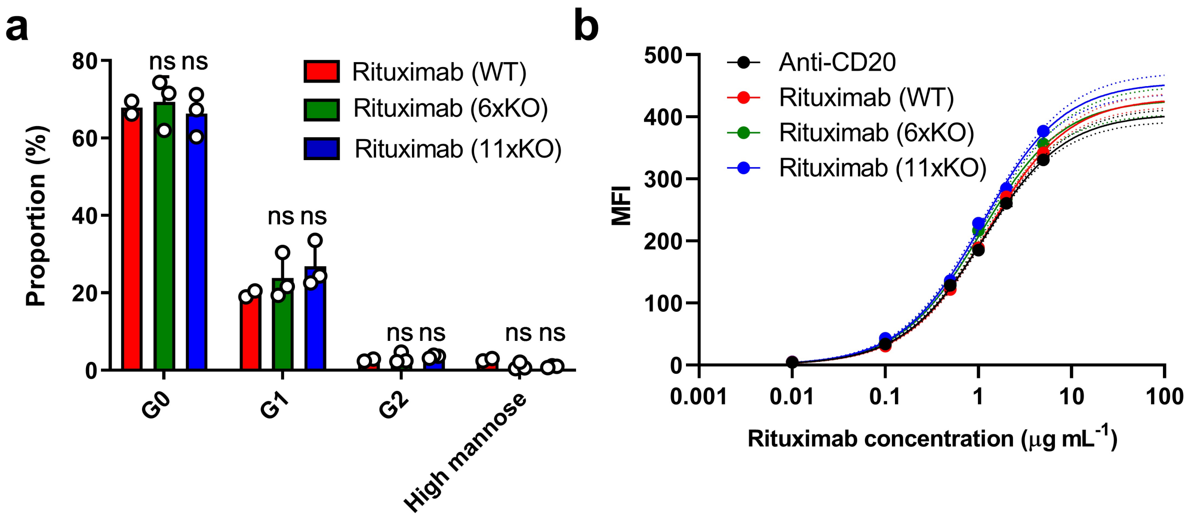

Supplementary Figure 6. The glycosylation profile and bioactivity of engineered cell line produced Rituximab are unaltered. **a** N-linked glycosylation structures of purified Rituximab from WT CHO (red bars), 6xKO (green bars), and 11xKO (blue bars) cell lines. G0, G1, G2 and high mannose represent G0+G0F, G1F+G1S1F, G2F+G2S1F+G2S2F, and M5+M7+M8 glycosylation structures, respectively. Data (6xKO and 11xKO) are presented as mean values +/- SD (*n* = 3 biologically independent experiments). Statistical analysis was performed using one-way ANOVA followed by Tukey's post-hoc test (ns = not significant). **b** The CD20-positive Ramos cell line was incubated with different concentrations of an anti-CD20 antibody (black), or Rituximab produced in wild-type (red), 6xKO (green), or 11xKO (blue) cell lines. After incubation with a fluorescently labelled secondary anti-human IgG antibody fragment and washing, fluorescence intensity was measured using flow cytometry. Data are presented as mean values +/- SD (*n* = 3 independent experiments). Curves were fitted using linear regression (one site; specific binding). Confidence intervals (95%) are shown as dotted lines. Only background levels of fluorescence were detected using the CD20-negative Jurkat cell line. MFI = mean fluorescence intensity. Source data are provided as a Source Data file.

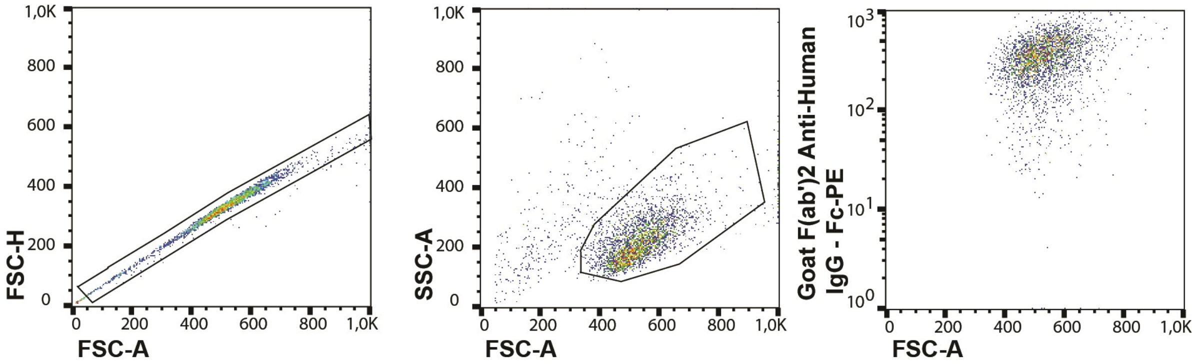

Supplementary Figure 7. Representative gating strategy used to obtain the mean fluorescence intensity (MFI) presented in Supplementary Figure 6b. In this example, Ramos cells were first incubated with 5 µg ml^{-1} Rituximab derived from WT CHO-S cells and then labelled with a secondary antibody fragment. Cells were gated for single cells (left panel), cells of interest (center panel) and the MFI of the phycoerythrin-labelled secondary anti-human-IgG F(ab')2 fragment was measured (right panel). Statistics are presented in Supplementary Data 3.

Text to Supplementary Table 1-2 and Supplementary Figure 8-10

Product quality is highly dependent on proper protein glycosylation[2,3]. To assess whether product quality had been affected by the series of knockouts, we used RNA-Seq to determine if there were any significant changes in secretory pathway subsystems and glycosylation gene sets in the 6x and 11x KO strains compared to WT. We first performed hypergeometric tests to determine if the differentially expressed genes (DEGs) caused by the different knockout cell lines were enriched for any secretory pathway modules (Supplementary Table 1) or glycosyltransferase gene sets (Supplementary Table 2). Of the 12 unique subsystems in the secretory pathway, only two, Golgi processing and protein folding, were modestly, but significantly affected in the 6xKO cell line on day 8. Protein processing within the Golgi involves modification of glycoproteins, however the lack of statistical significance (FDR <0.05) for any of the glycosylation gene sets indicates that glycotransferases were not affected by the series of gene knockouts. To further understand what cellular response might be impacted, we performed Gene set enrichment analysis (GSEA) to identify pathways (KEGG and REACTOME) and Gene Ontology terms over-represented in the list of DEGs. Our results show that the knockouts mainly impact REACTOME pathways (Supplementary Figure 8) related to six major categories of pathways. Three are up-regulated pathways: 1) cell cycle, 2) DNA repair, 3) Gene expression, and the other three are down-regulated pathways: 4) immune system, 5) signaling transduction, and 6) developmental biology. The enriched KEGG pathways (Supplementary Figure 9) and Gene Ontology terms (Supplementary Figure 10) showing similar functional categories. Together, the absence of glycosylation pathways in the GSEA enrichment combined with the insignificant changes in glycotransferases according to the hypergeometric tests suggest that the KOs have not altered protein glycosylation capabilities, which is further supported by the lack of changes in glycosylation (Supplementary Figure 6a).

	6x			11x		
	Day4	Day6	Day8	Day4	Day6	Day8
**COPI**	1.000	1.000	1.000	1.000	1.000	1.000
**COPII**	1.000	1.000	1.000	1.000	1.000	1.000
**Clathrin vesicles**	1.000	1.000	1.000	1.000	1.000	1.000
**Dolichol pathway**	1.000	1.000	1.000	1.000	1.000	1.000
**ER glycosylation**	1.000	1.000	1.000	1.000	1.000	1.000
**ERAD**	1.000	1.000	1.000	1.000	1.000	1.000
**GPI biosynthesis**	1.000	1.000	1.000	1.000	1.000	1.000
**GPI transfer**	1.000	1.000	1.000	1.000	1.000	1.000
**Golgi processing**	1.000	0.535	**0.034**	1.000	1.000	0.373
**Protein Folding**	1.000	1.000	**0.034**	1.000	1.000	1.000
**SV**	1.000	1.000	1.000	1.000	1.000	1.000
**Translocation**	1.000	1.000	1.000	1.000	1.000	1.000

Supplementary Table 1. Hypergeometric enrichment results of secretory pathway subsystems. After Benjamini-Hochberg false discovery correction, a subsystem is deemed significant if it has an adjusted p-value less than 0.05. Of the 12 unique subsystems in the secretory pathway, only two (Golgi processing and protein folding) were significantly affected in the 6xKO cell line on day 8. Statistical analysis was performed using one-tailed Fisher's exact test.

		6x			11x		
		**Day4**	**Day6**	**Day8**	**Day4**	**Day6**	**Day8**
**Keratan-sulfate**	Differentially expressed genes	1003	1280	1896	953	1233	1139
	Genes in glycotransferase gene set	12	12	12	12	12	12
	Differntially expressed glycotransferase genes	1	2	3	2	3	3
	FDR	1.000	0.921	0.616	0.979	0.476	0.380
**N-glycan**	Differentially expressed genes	1003	1280	1896	953	1233	1139
	Genes in glycotransferase gene set	27	27	27	26	27	27
	Differntially expressed glycotransferase genes	0	2	3	2	2	1
	FDR	1.000	0.921	0.979	0.979	0.897	1.000
**Chondroitin-sulfate**	Differentially expressed genes	1003	1280	1896	953	1233	1139
	Genes in glycotransferase gene set	9	9	9	9	9	9
	Differntially expressed glycotransferase genes	1	1	1	1	1	1
	FDR	1.000	0.921	0.979	0.979	0.8971	0.964
**O-glycan mucin**	Differentially expressed genes	1003	1280	1896	953	1233	1139
	Genes in glycotransferase gene set	16	16	16	15	15	16
	Differntially expressed glycotransferase genes	1	2	4	1	1	2
	FDR	1.000	0.921	0.616	0.979	0.8971	0.964
**Heparan-sulfate**	Differentially expressed genes	1003	1280	1896	953	1233	1139
	Genes in glycotransferase gene set	12	12	12	12	12	12
	Differntially expressed glycotransferase genes	0	0	0	1	1	0
	FDR	1.000	1.000	1.000	0.979	0.897	1.000
**O-glycan notmucin**	Differentially expressed genes	1003	1280	1896	953	1233	1139
	Genes in glycotransferase gene set	21	21	21	21	21	21
	Differntially expressed glycotransferase genes	0	2	2	1	2	2
	FDR	1.000	0.921	0.979	0.979	0.897	0.964
**GPI-anchor**	Differentially expressed genes	1003	1280	1896	953	1233	1139
	Genes in glycotransferase gene set	20	20	20	20	20	20
	Differntially expressed glycotransferase genes	0	0	0	0	0	0
	FDR	1.000	1.000	1.000	1	1.000	1.000
**All glycotransferases**	Differentially expressed genes	1003	1280	1896	953	1233	1139
	Genes in glycotransferase gene set	100	100	100	98	99	100
	Differntially expressed glycotransferase genes	3	7	10	4	6	6
	FDR	1.000	0.921	0.979	0.980	0.897	1.000

Supplementary Table 2. Hypergeometric enrichment results of glycosylation gene sets. After Benjamini-Hochberg false discovery correction, a glycosylation gene set is deemed significant if it has an FDR adjusted p value less than 0.05. There was no significant enrichment for any of the glycotransferase classes. Statistical analysis was performed using one-tailed Fisher's exact test.

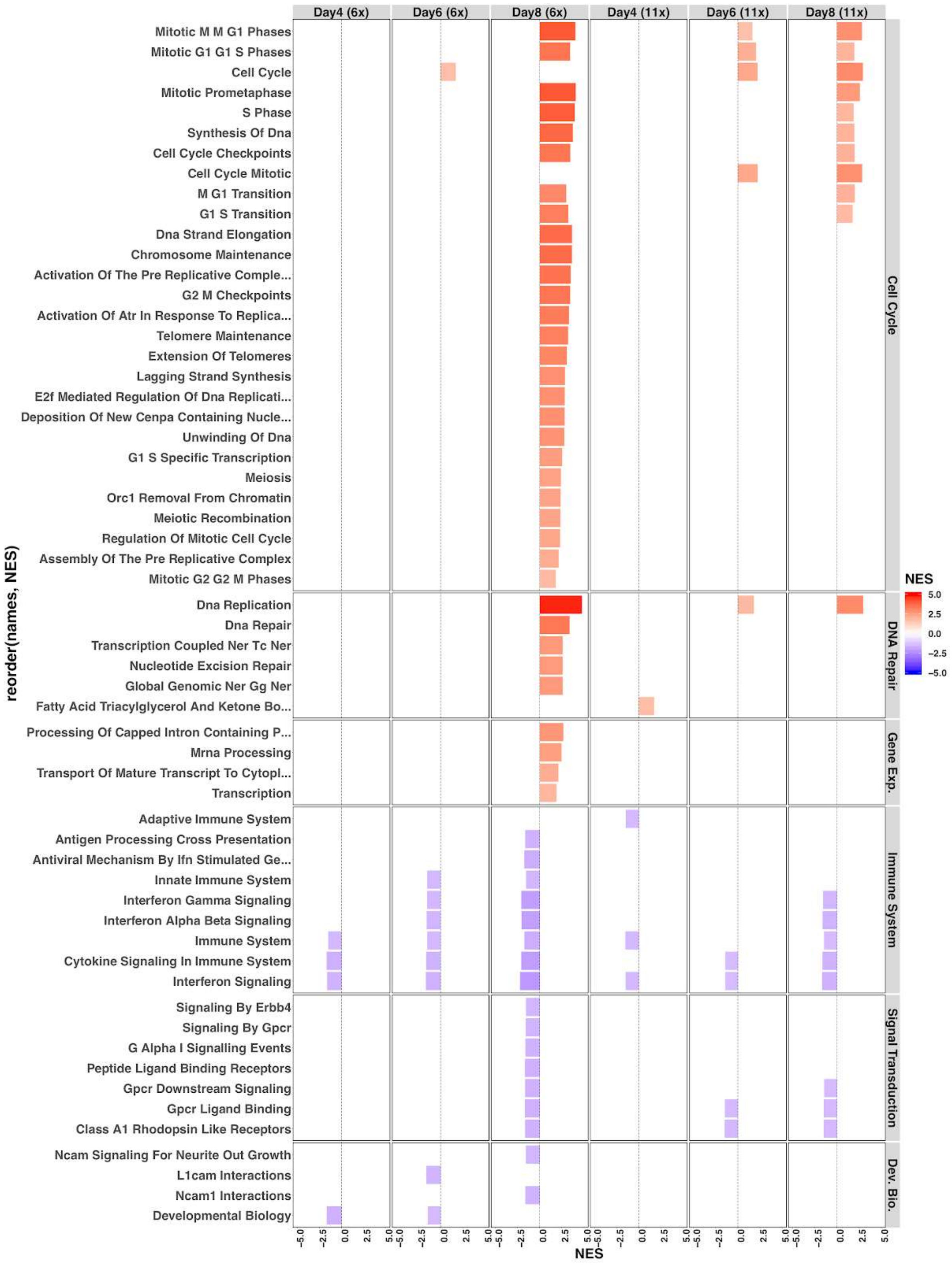

Supplementary Figure 8. Enriched REACTOME pathways. Reactome pathways and hallmark gene sets were enriched for differentially expressed genes following 6x/11x KOs in CHO cells.

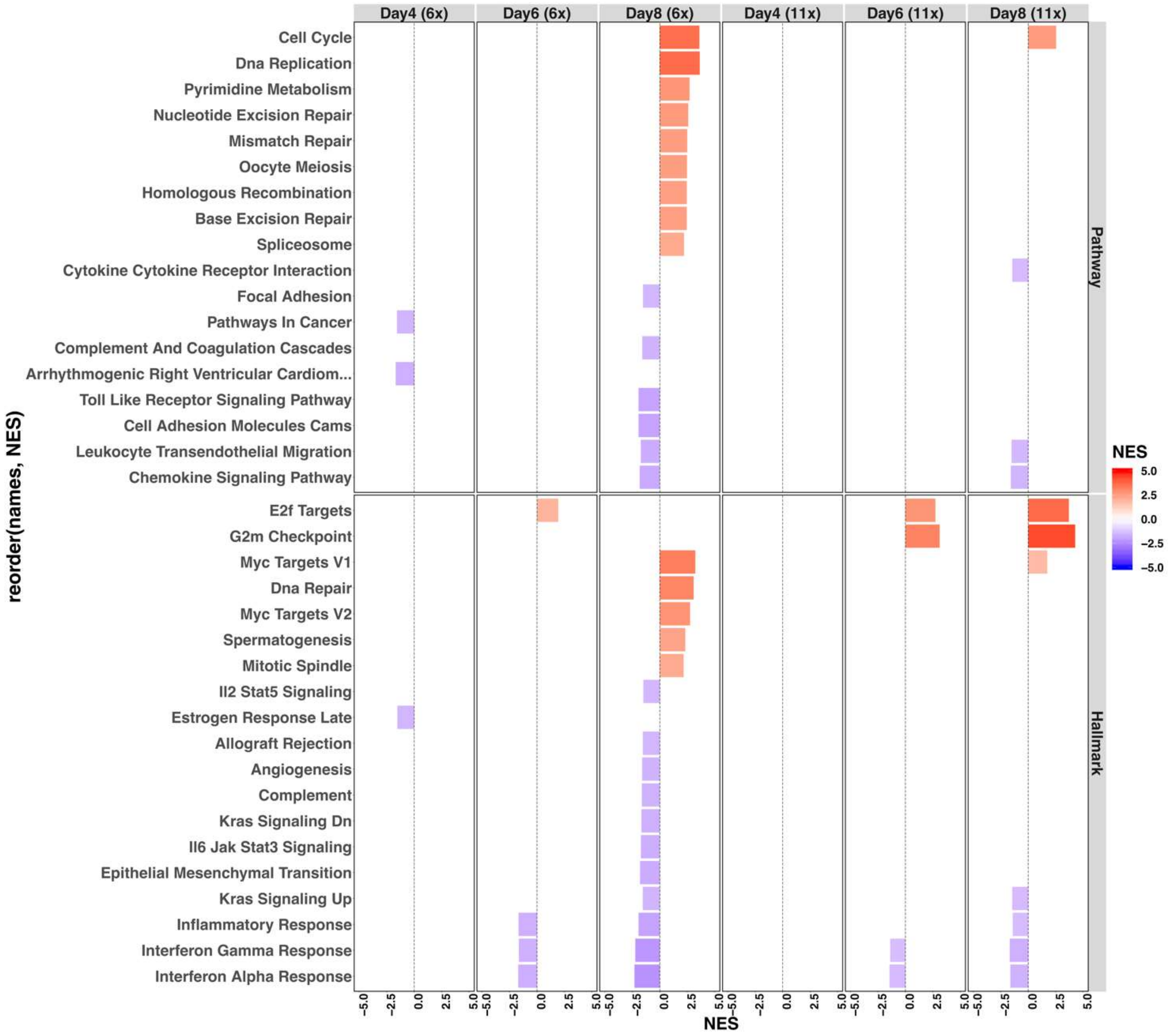

Supplementary Figure 9. Enriched KEGG pathways. KEGG pathways and hallmark gene sets were enriched for differentially expressed genes following 6x/11x KOs in CHO cells.

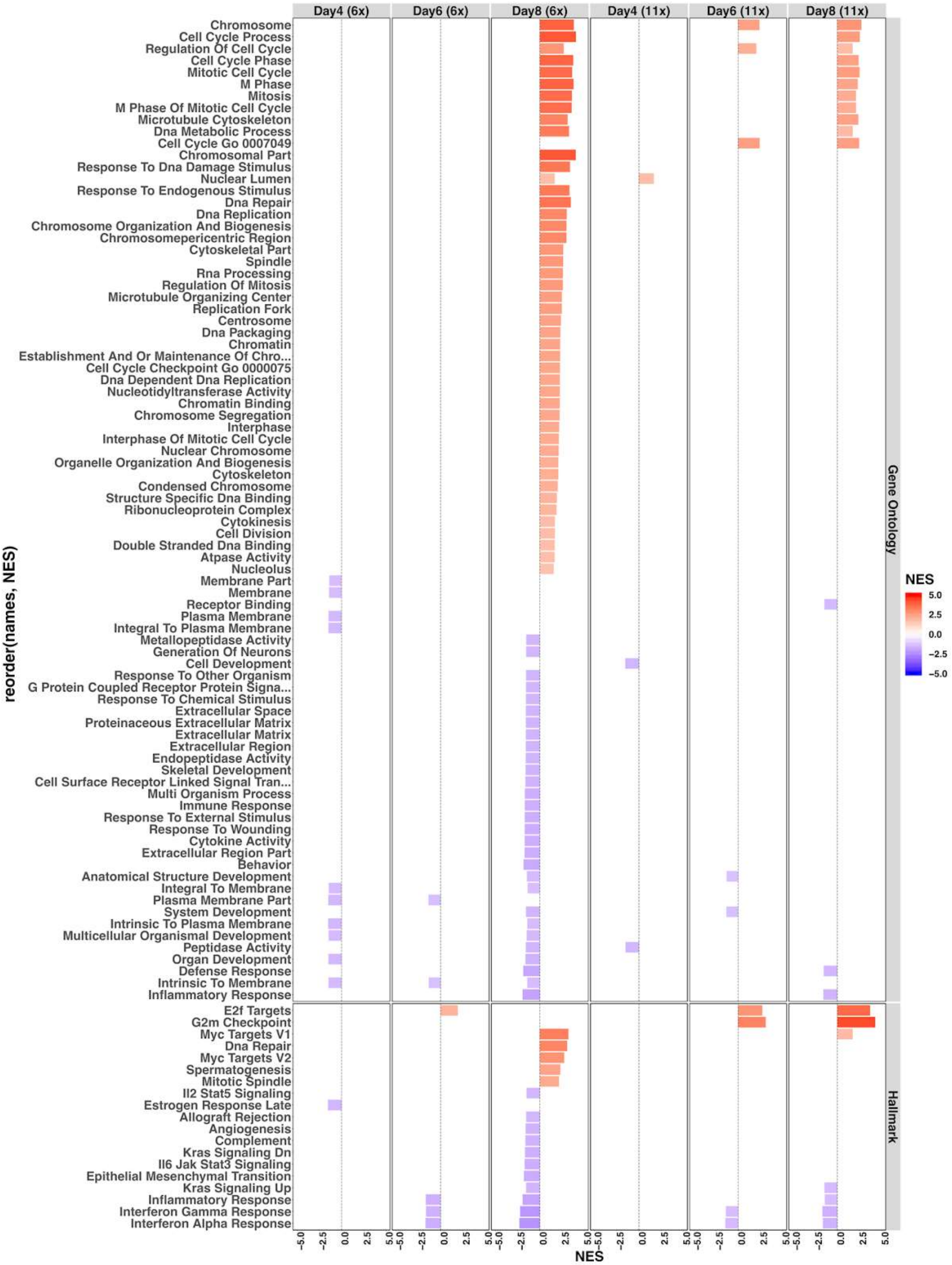

Supplementary Figure 10. Enriched Gene Ontology terms. GO terms and hallmark gene sets were enriched for differentially expressed genes following 6x/11x KOs in CHO cells.

Supplemental references

1. Amann, T. *et al.* Glyco-engineered CHO cell lines producing alpha-1-antitrypsin and C1 esterase inhibitor with fully humanized N-glycosylation profiles. *Metab. Eng.* **52**, 143–152 (2019).
2. Sola, R. J. & Griebenow, K. Effects of glycosylation on the stability of protein pharmaceuticals. *Journal of Pharmaceutical Sciences* **98**, 1223–1245 (2009).
3. del Val, I. J., Kontoravdi, C. & Nagy, J. M. Towards the implementation of quality by design to the production of therapeutic monoclonal antibodies with desired glycosylation patterns. *Biotechnol. Prog.* **26**, 1505–1527 (2010).
