# Supplementary material for: Multiplex secretome engineering enhances recombinant protein production and purity
Source: Nat Commun. 2020 Apr 20;11:1908. doi: 10.1038/s41467-020-15866-w (PMC7170862; doi:10.1038/s41467-020-15866-w)
Supplement: Supplementary file 6 — Description of Additional Supplementary Files [file 41467_2020_15866_MOESM6_ESM.pdf]

## Description of Additional Supplementary Files

**Title:** Supplementary Data 1

**Description:** Proteomic analysis of WT CHO-S HCPs provided as a spreadsheet file. UniProt identifiers, protein names and relative abundance of the 200 most abundant proteins found in a CHO-S supernatant are shown. The targets addressed in this study are indicated in bold. Abundance values are the mean of technical triplicates.

**Title:** Supplementary Data 2

**Description:** Spreadsheet containing all primer names and sequences used in this study.

**Title:** Supplementary Data 3

**Description:** Spreadsheet containing flow cytometry raw data and statistics.
