# Supplementary material for: Multiplex secretome engineering enhances recombinant protein production and purity
Source: Nat Commun. 2020 Apr 20;11:1908. doi: 10.1038/s41467-020-15866-w (PMC7170862; doi:10.1038/s41467-020-15866-w)
Supplement: Supplementary file 7 — Reporting Summary [file 41467_2020_15866_MOESM7_ESM.pdf]

nature research

Corresponding author(s): Stefan Kol
Nathan E. Lewis

Last updated by author(s): Mar 20, 2020

Reporting Summary

Nature Research wishes to improve the reproducibility of the work that we publish. This form provides structure for consistency and transparency in reporting. For further information on Nature Research policies, see Authors & Referees and the Editorial Policy Checklist .

Statistics

For all statistical analyses, confirm that the following items are present in the figure legend, table legend, main text, or Methods section.

n/a	Confirmed	
☐	☒	The exact sample size (*n*) for each experimental group/condition, given as a discrete number and unit of measurement
☐	☒	A statement on whether measurements were taken from distinct samples or whether the same sample was measured repeatedly
☐	☒	The statistical test(s) used AND whether they are one- or two-sided<br>*Only common tests should be described solely by name; describe more complex techniques in the Methods section.*
☒	☐	A description of all covariates tested
☐	☒	A description of any assumptions or corrections, such as tests of normality and adjustment for multiple comparisons
☐	☒	A full description of the statistical parameters including central tendency (e.g. means) or other basic estimates (e.g. regression coefficient) AND variation (e.g. standard deviation) or associated estimates of uncertainty (e.g. confidence intervals)
☐	☒	For null hypothesis testing, the test statistic (e.g. *F*, *t*, *r*) with confidence intervals, effect sizes, degrees of freedom and *P* value noted<br>*Give P values as exact values whenever suitable.*
☒	☐	For Bayesian analysis, information on the choice of priors and Markov chain Monte Carlo settings
☒	☐	For hierarchical and complex designs, identification of the appropriate level for tests and full reporting of outcomes
☒	☐	Estimates of effect sizes (e.g. Cohen's *d*, Pearson's *r*), indicating how they were calculated

Our web collection on statistics for biologists contains articles on many of the points above.

Software and code

Policy information about availability of computer code

Data collection	Flow cytometry data was collected using FlowJo v10 (BD Biosciences)
Data analysis	Statistical analysis was done using GraphPad Prism 8.4, modelling of protein secretion was performed using MATLAB 2018B [https://github.com/LewisLabUCSD/CHOSecretoryKO], liquid chromatography data was analyzed with Progenesis QI software v3.0, glycans were quantified using Thermo Xcalibur software v4.1. RNA-seq analysis was performed using FastQC v0.11, Trimmomatic v0.39, STAR v2.7.0a, HTSeq v0.11.3, DESeq2 v1.26.0 Gene set enrichment analysis (GSEA) was performed using the Broad Institute GSEA software v3.0.0.

For manuscripts utilizing custom algorithms or software that are central to the research but not yet described in published literature, software must be made available to editors/reviewers. We strongly encourage code deposition in a community repository (e.g. GitHub). See the Nature Research guidelines for submitting code & software for further information.

Data

Policy information about availability of data

All manuscripts must include a data availability statement. This statement should provide the following information, where applicable:

- Accession codes, unique identifiers, or web links for publicly available datasets
- A list of figures that have associated raw data
- A description of any restrictions on data availability

Raw RNAseq data that support the findings of this study have been deposited at the Gene Expression Omnibus and Short Read Archive with the accession number GSE144624 [https://www.ncbi.nlm.nih.gov/geo/query/acc.cgi?acc=GSE144624]. The authors declare that all other data supporting the findings of this study are available within the paper and its supplementary information files. The source data for figures 1-5, table 1, and supplementary figures 1-4 and 6 are provided as a source data file.

Field-specific reporting

Please select the one below that is the best fit for your research. If you are not sure, read the appropriate sections before making your selection.

[x] Life sciences [ ] Behavioural & social sciences [ ] Ecological, evolutionary & environmental sciences

For a reference copy of the document with all sections, see nature.com/documents/nr-reporting-summary-flat.pdf

Life sciences study design

All studies must disclose on these points even when the disclosure is negative.

Sample size	Sample size choice was not predetermined by a statistical method.
Data exclusions	No data were excluded from analysis
Replication	We confirm that all attempts at replication were successful. Statistical parameters including the exact value of n, p values, and the types of the statistical tests are reported in the figures and corresponding figure legends. Statistical analysis was carried out using Prism 8.4 (GraphPad Software). Statistical analysis was conducted on data from three or more independent experimental replicates. Data distribution was assumed to be normal, but this was not formally tested. Comparisons between groups were planned before statistical testing and target effect sizes were not predetermined. Error bars displayed on graphs represent the mean+/-SD of at least three independent experiments. Most experiments report technical replicates, whereas biological variability was addressed in the clonal variation experiment. *p<0.05, **p<0.01, ***p<0.001, and ****p < 0.0001 were considered significant.
Randomization	No randomization method was used
Blinding	Genome editing and all assays were run by a supporting lab technicians who were not informed on the study nor expected outcomes. Analysis was performed unblind.

Reporting for specific materials, systems and methods

We require information from authors about some types of materials, experimental systems and methods used in many studies. Here, indicate whether each material, system or method listed is relevant to your study. If you are not sure if a list item applies to your research, read the appropriate section before selecting a response.

Materials & experimental systems

n/a	Involved in the study	
[ ]	[x]	Antibodies
[ ]	[x]	Eukaryotic cell lines
[x]	[ ]	Palaeontology
[x]	[ ]	Animals and other organisms
[x]	[ ]	Human research participants
[x]	[ ]	Clinical data

Methods

n/a	Involved in the study	
[x]	[ ]	ChIP-seq
[ ]	[x]	Flow cytometry
[x]	[ ]	MRI-based neuroimaging

Antibodies

Antibodies used	Primary: Human CD20 (Rituximab) MAb (Clone Hu2) MAB9575. Secondary: Goat F(ab')2 Anti-Human IgG - Fc (PE), pre-adsorbed (ab98596). Goat anti-Human IgG Fc Secondary Antibody, FITC (H10001C)
Validation	https://www.rndsystems.com/products/human-cd20-rituximab-mab-clone-hu2-hu2_mab9575. https://www.abcam.com/goat-fab2-human-igg-fc-pe-pre-adsorbed-ab98596.html. https://www.thermofisher.com/antibody/product/Goat-anti-Human-IgG-Fc-Secondary-Antibody-Polyclonal/H10001C

Eukaryotic cell lines

Policy information about cell lines

Cell line source(s)	CHO-S cell line, Thermo Fisher. Jurkat cell line, DSMZ, ACC 282. Ramos cell line, DSMZ, ACC 603.
Authentication	Original CHO-S cell line was banked according to cGMP rules. All cell lines from DSMZ have been thoroughly tested and athenticated.
Mycoplasma contamination	All new cell lines were tested negative for Mycoplasma infection and were kept in quarantine until confirmed.

Commonly misidentified lines (See ICLAC register)	none

Flow Cytometry

Plots

Confirm that:

- [x] The axis labels state the marker and fluorochrome used (e.g. CD4-FITC).
- [x] The axis scales are clearly visible. Include numbers along axes only for bottom left plot of group (a 'group' is an analysis of identical markers).
- [x] All plots are contour plots with outliers or pseudocolor plots.
- [x] A numerical value for number of cells or percentage (with statistics) is provided.

Methodology

Sample preparation	Ramos cells (2x105 cells per well; DSMZ, ACC 603) were blocked with PBS containing 5% BSA for 10 minutes and incubated with 100 µL of the Rituximab dilutions for 1 h at 4°C. Cells were washed 3 times with PBS and subsequently incubated for 30 min at 4° C with 5 ug/ml of phycoerythrin-conjugated goat F(ab') anti-human IgG (Abcam, Cambridge, UK) as secondary antibody. After washing the cells 3x with PBS, binding was quantified in triplicate by flow cytometry using MACSQuant analyzer 10 VYB (Miltenyi Biotec, Bergisch Gladbach, Germany). Jurkat cells were used as CD20 negative cell line and a Rituximab biosimilar antibody (R&D systems, Minneapolis, MN, USA) was included as reference anti-CD20 antibody at the indicated dilutions.
Instrument	MACSQuant VYB (Miltenyi Biotec)
Software	FlowJo v10 (BD Biosciences)
Cell population abundance	no cell sorting was performed
Gating strategy	FSC-H/FSC-A was the initial gating for singlets, then FSC-A/SSC-A for cells of interest, then the mean fluorescence intensity was measured.

- [x] Tick this box to confirm that a figure exemplifying the gating strategy is provided in the Supplementary Information.
